# Valorization of Colombian fique (*Furcraea bedinghausii*) for production of cellulose nanofibers and its application in hydrogels

**DOI:** 10.1038/s41598-020-68368-6

**Published:** 2020-07-15

**Authors:** Marcelo A. Guancha-Chalapud, Jaime Gálvez, Liliana Serna-Cock, Cristobal N. Aguilar

**Affiliations:** 1 National Center for Technical Assistance to Industry (ASTIN), Servicio Nacional de Aprendizaje – SENA, Medellín, Colombia; 2 Faculty of Engineering and Administration, Universidad Nacional de Colombia Campus Palmira, Palmira, Colombia; 3 Bioprocesses and Bioproducts Research Group. Food Research Department, School of Chemistry. Universidad Autónoma de Coahuila, Saltillo, Mexico

**Keywords:** Chemistry, Engineering, Materials science

## Abstract

Cellulose nanofibers were obtained from the Colombian fique (*Furcraea bedinghausii*) and Acrylic hydrogels (H) and reinforced acrylic hydrogels with fique nanofibres (HRFN) were synthesized, using the solution polymerization method. The extraction was carried out using a combined extraction method (chemical procedures and ultrasound radiation). The raw material (NAT-F), bleached fibers (B-F), hydrolyzed fibers and fibers treated with ultrasound (US-F) were characterized by infrared spectroscopy (FTIR) and thermal stability analysis; also, in order to have a comparison criterion, a commercial microcrystalline cellulose sample (CC) was analyzed, which demonstrated the extraction of fique cellulose. The surface morphology of the NAT-F and the B-F was determined by scanning electron microscopy and the average particle size of the nanofibers was made through transmission electron microscopy. In H y HRFN the strain percent and compression resistance (Rc) were measured. The fique nanofibers showed diameter and length averages of 25.2 ± 6.2 nm and 483.8 ± 283.2 nm respectively. Maximum degradation temperature was 317 °C. HRFN presented higher compression resistance (16.39 ± 4.30 kPa) and this resistance was 2.5 greater than the resistance of H (6.49 ± 2.48 kPa). The results indicate that fique lignocellulosic matrix has potential application for obtaining polymeric type composite materials.

## Introduction

Hydrogels are polymers with a crisscrossed three-dimensional structure which allows to absorb, store and release water molecules^1^. Its application is oriented to the manufacture of personal hygiene products, medical^2,3^, environmental^4–6^ and agricultural applications^7,8^. Hydrogels for application in agriculture are usually synthesized based on acrylates or similar monomers (acrylamide, methyl methrillate among others), because cross-linked polymers with high absorption capacity and bloating rate are obtained^1,9^ and low toxicity^10,11^. One of the main limitations of hydrogels for agricultural applications is their low mechanical strength^10,12,13^. The pressure exerted by the plant and the soil layer on the hydrogel influence the loss of swelling capacity, elasticity and stiffness^14,15^. To maintain polymer elasticity, long-chain molecules and adequate interbreeding are needed to dissipate external mechanical stresses on the hydrogel^10,13,16^. To improve mechanical properties, manometric-sized reinforcement materials such as: inorganic nanoclafers, carbon nanotubes, nanoparticles of inorganic compounds are usually used^11,17^. However, one of the disadvantages of using reinforcement materials is the decrease in water absorption capacity^18^. As consequence, an alternative is the use of micro-scale cellulose and nano as a reinforcement material in hydrogels^19^. Several studies show that the addition of cotton nanofibers^9,20^, chitosan^21^, wood^10,12,22^ into acrylic base hydrogels, increases, mechanical strength and swelling capacity due to increased surface area, elastic character of fibers and the presence of hydroxyl groups within the polymer matrix^22–24^. The addition of nanofibers in hydrogels increases compression resistance by up to 50%^14,22^, and the ability to recover its original shape (elasticity) from the application of external forces, up to 80% compared to its original height^11,14^.

For the use of cellulose nanofibers as reinforcing agents, cellulose sources that are available, sustainable and inexpensive should be considered, therefore, an alternative is the use of agro-industrial waste^25^. Agricultural activity generates a large amount of crop waste, including fique^26^. The fique agribusiness in Colombia focuses on the production of fibers^27^, however, the fibers represent only 4% of the leaves, the remaining 96% corresponds to waste generated in the process, such as juice (70%) bagasse or cabuyasa (30%)^27,28^. Consequently, fique lignocellulosic residues are an alternative source for nanofiber isolation. Fique fibers are characterized by their high cellulose content (50–74%), specific strength, biodegradability^29^, low density (1.5 g cm^−3^ compared to fiberglass 2.5 g cm^−3^)^30^, and is considered one of the most tensile resistant (511–635 MPa) compared to other natural fibers^31^.

Chemical pretreatment of cellulosefibers is essential to improving nanofibrillation and decreasing energy consumption^25^. These pretreatments are necessary because lignin and hemicelulose coat^15,16,26,27^ cellulose chains and lead to different qualities and morphologies of nanofiber^25^. To break the amorphous region of the chains and isolate cellulose nanofibers, several methods have been used since 2008 including acid hydrolysis, enzyme processes, oxidation, high-pressure homogenization, high-intensity ultrasound and steam blasting processes^32^. Recently new approaches to pretreatment have been developed that do not hydrolyze or oxidize the cellulose fibers, which include the use of deep eutectic solvent, NaOH swelling, Imidazole, Carboxymethyl cellulose adsorption, but their detailed mechanisms remain to be investigated^33^.

In the isolation of nanofibers the use of concentrated chemicals or mechanical methods for long periods of time degrade cellulose decreasing the degree of polymerization of chains^34^. The use of combined methods decreases cellulose degradation and optimizes morphology and aspect ratio^32^. To increase the yields in obtaining nanocellulose is required to separate cellulose chains from the crystalline part, one of the methods used for this purpose is the ultrasound technique. The transfer of ultrasonic energy to the cellulose chains generated by the cavitation process, is similar to the energy to break a hydrogen bond (100 kJ/mol), therefore, when applying this technique can disintegrate cellulose fibers from the crystalline part, of micrometric size to nanometric size^35,36^.

The objectives of this paper were to obtain fique nanofibers using chemical methods combined with ultrasound radiation and to evaluate the effect of fique nanofibers as reinforcing material in acrylic hydrogels. Morover, fibers and nanofibers study was made using infrared analysis (FITR), scanning electron microscopy (SEM), transmission electron microscopy (TEM) and thermogravimetric analysis (TGA); additionally, the strain and resistance to compression of the reinforced hiydrogels are discussed.

## Results and discussion

### Characterization of raw material (NAT-F) and bleached fibers (B-F)

Cellulose and lignin contents of NAT-F were 45, 42 and 21.34% respectively, and B-F were 91.92% and 4.14% respectively. The fiber bleaching process was efficient as it managed to eliminate more than 80% of the lignin present in NAT-F. These results were similar to the results reported by Ovalle et al.^26^ where after the delignification process the lignin content decreased from 23.3 to 2.8% and the cellulose content increased from 52.3 to 83.6%.

**Figure 1:**
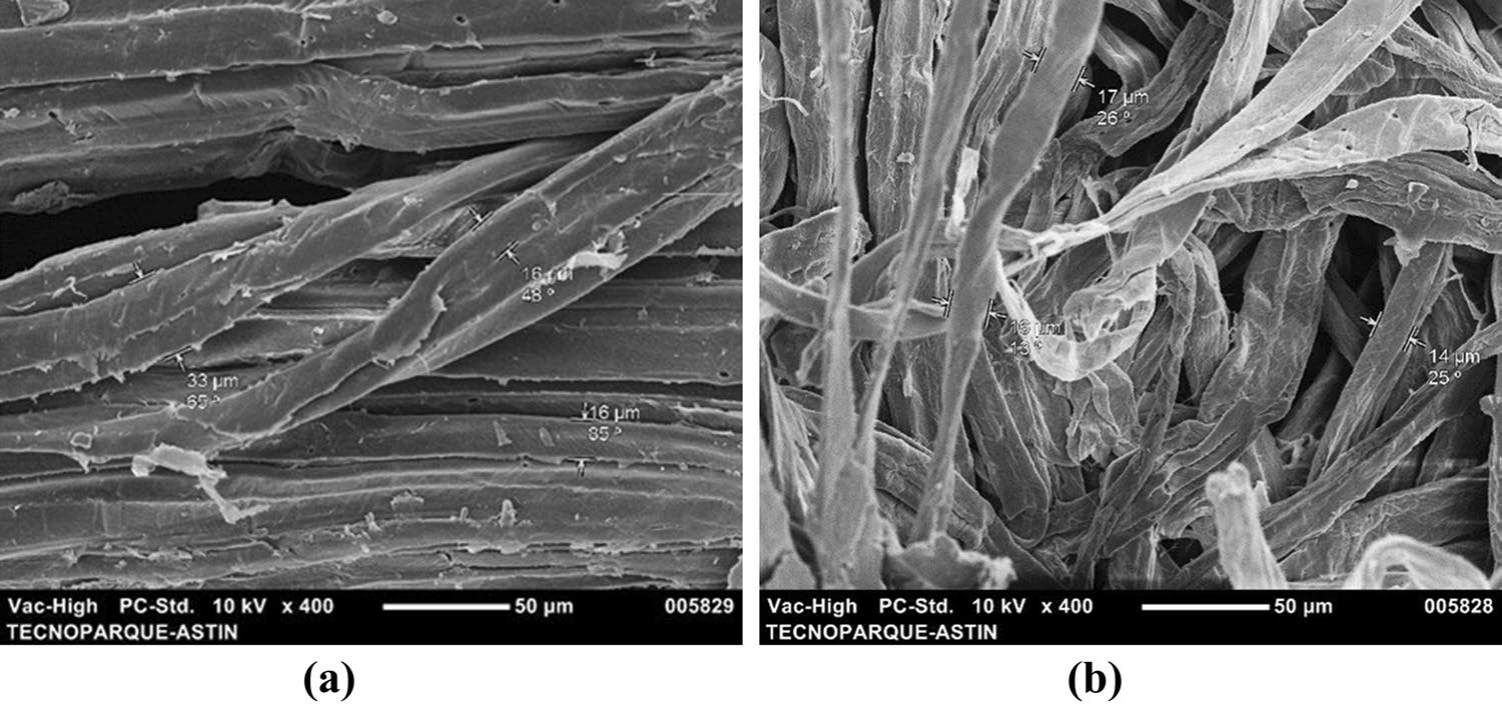
SEM micrographs (**a**) native fique (NAT-F), (**b**) bleached fibers (B-F).

SEM micrographs were performed to have the particle size of the different products obtained during the nanofibrilation process from fique. The Fig. 1 shows the SEM micrographs of NAT-F and B-F. NAT-F (Fig. 1a) appears as agglutinated fibers, with a rigid appearance and an approximate diameter of 34.2 μm. B-F (Fig. 1b) has a surface roughness and the fiber diameter ranged from 9 to 21.4 μm. According to Candra et al.^37^ lignocellulosic materials without any treatment do not show homogeneity due to the presence of compounds such as lignin, hemicellulose, pectin, among others, which act as binders, preventing defibrillation of cellulose chains. Additionally, roughness in bleached fique fiber is due to nanofibrils which are connected through hydrogen bonds, to form larger units of microfibrils^36,37^.

### FITR analysis

**Figure 2:**
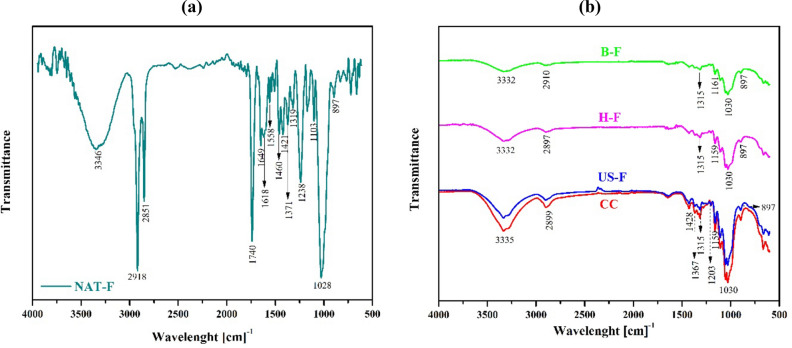
(**a**) Infrared spectrum (FITR) of untreated native fique fibers (NAT-F), (**b**) infrared spectra of bleached fibers (B-F), hydrolyzed fibers (H-F), fibers treated with ultrasound (US-F). Commercial.

Infrared spectrum of the NAT-F is shown in Fig. 2a, b shows the FTIR spectra of B-F, H-F, US-F and CC.

Infrared spectrum of the NAT-F shows two absorption zones. The absorption band that was obtained from 3346 cm^−1^ for the raw material corresponds to hydroxyl groups (–OH) and the band at 2,918 cm^−1^ corresponds to C–H stretch present in cellulose, hemicellulose and lignin^38^. The carbonyl group band appears at 1,740 cm^−1^, and its correspond to bond vibration present in the ester and carboxylic groups (–COOCH3 and –COOH) characteristic of hemicellulose, ferulic and p-coumaric acids, which belongs to lignin^38,39^. The band at 1,238 cm^−1^ corresponds to –C–O–C– bond vibration present in the bonds between aromatic ring and methoxy groups in lignin^40^. The bands at 1,649, 1,460 and 1,421 cm^−1^ for raw material, corresponds to the C=C bond stretching present in aromatic rings of lignin^37,38,40,41^. Finally, at 1,028 cm^−1^ for raw material refers to C–O–C bond stretching of β-1,4-glycosidic ring bonds among d-glucose units in cellulose^37^.

To remove the lignin, hemicelluloses, waxes and other components was applied an alkali treatment (delignification) and to remove completely the lignin the bleaching treatments was performed, obtaining B-F. The FTIR spectra of B-F, H-F, US-F and CC. B-F FTIR spectrum shows the disappearance of several peaks (1,740, 1,238, 1,649, 1,460 y 1,421 cm^−1^) caused by the removal of lignin and hemicellulose due to chemical treatment. It should be mentioned that, in addition, an increasing in peaks intensity around 1,030, 3,332 and 2,900 cm^−1^, indicate the presence of cellulose and become more pronounced in the H-F and US-F spectra. It is observed, in addition, in B-F spectrum the appearance of peaks in 1,160, 1,315 and 897 cm^−1^. Additionally, these peaks become more intense due to the effect of acid treatment followed by treatment with ultrasound radiation. The presence of peak at 1,428 cm^−1^ and an increasing in peaks intensity around 1,367 and 1,315 cm^−1^ in US-F spectrum is in line with that reported by Avolio et al.^42^ and Candra et al.^37^, where is indicated that acute signals around 1,426 and 897 cm^−1^, 1,370 and 2900 cm^−1^ reflect the crystalline band of cellulose. In addition, Fan et al. ^43^ report that sharp peaks around 1,420 and 894 cm^−1^ indicate the presence of crystalline structures, a widening of these bands reflects a more disordered structure that would correspond to amorphous cellulose.

### Thermogravimetric analysis

**Figure 3:**
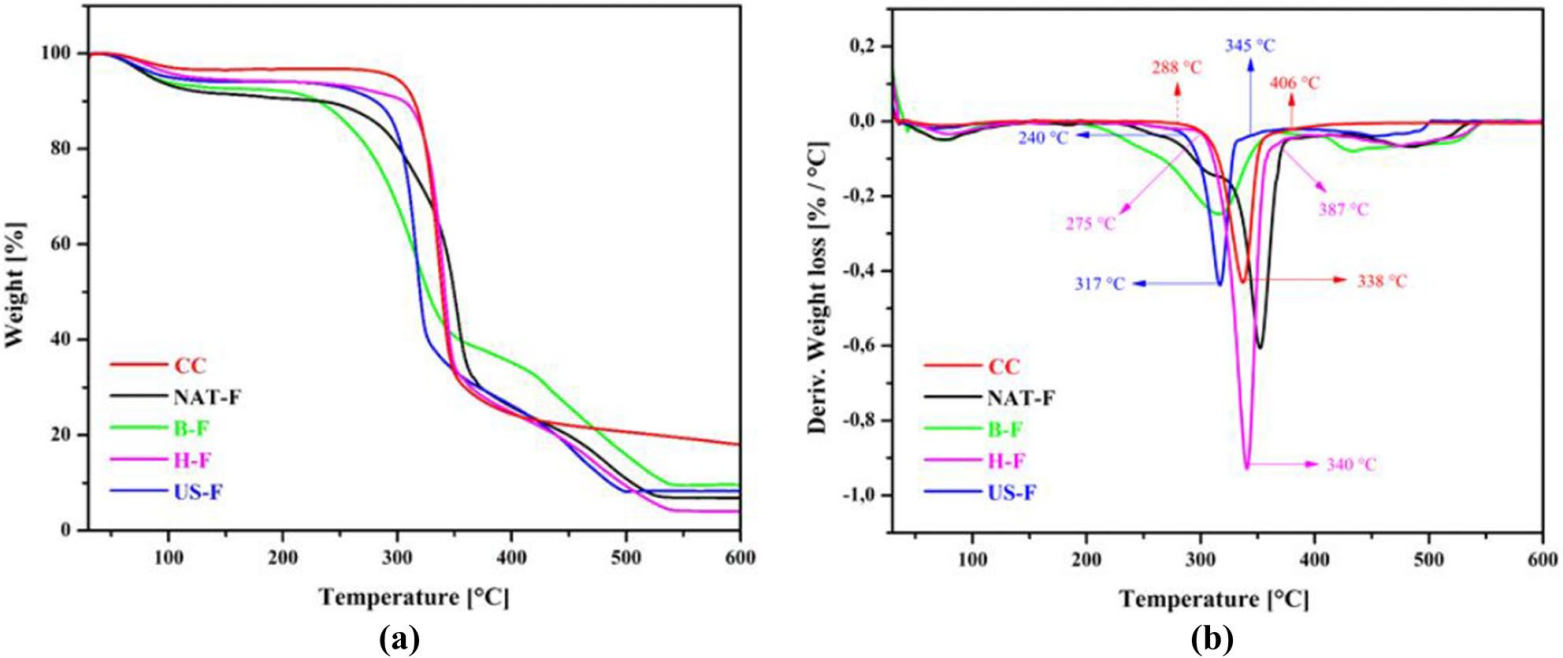
(**a**) Thermogravimetric analysis of fique fibers: native untreated fibers (NAT-F), bleached fibers (B-F), hydrolyzed fibers (H-F), fibers treated with ultrasound (US-F). Commercial cellulose (CC). (**b)** Derivative thermogravimetric analysis of fique fibers: native untreated fibers (NAT-F), bleached fibers (B-F), hydrolyzed fibers (H-F), fibers treated with ultrasound (US-F). Commercial cellulose (CC).

The thermogravimetric (TG) results (Fig. 3) are summarized in Table 1.

**Table 1:** Thermal parameters obtained TGA analysis of: untreated native fique fibers (NAT-F), bleached fibers (B-F), hydrolyzed fibers (H-F), fibers treated with ultrasound (US-F). Commercial cellulose (CC).

	T_0_ (°C)	T_max_ (°C)	T5% (°C)	T10% (°C)
NAT-F	228	352	83	200
B-F	192	316	88	229
H-F	292	340	115	303
US-F	250	317	107	278
CC	288	337	295	314

A thermal stability analysis was performed to demonstrate the efficiency of bleaching, hydrolysis acid and ultrasound treatment treatments (Fig. 3). Three stages of degradation were observed: the first, below 250 °C, the second between 250 °C and 400 °C and the third above 400 °C (Fig. 3a). For all the products was found a small weight loss corresponding to the removal of moisture in the range of 25–150 °C, due to water evaporation process or loss of low molecular weight compounds. In all cases, the cellulosic materials shown maximum temperature degradation from 316 to 352 °C, and a third less pronounced peak between 400 and 500 °C because of the lignin residues in the raw material and the delignificated cellulose. The initial degradation temperatures were found to be around 228, 192, 292, 250 and 288 °C for the NAT-F, B-F, H-F, US-F and CC, respectively. The initial degradation temperatures of H-F (292 °C) and US-F (250 °C) were appreciably increased compared with the NAT-F temperature (228 °C) due to the partial removal of hemicelluloses, lignin and pectin by delignification and bleaching process (Fig. 3b). B-F had a shoulder between 200 and 300 °C and a slightly pronounced peak between 400 and 500 °C. This shows that treatment with hydroxide and chlorite did not completely eliminate hemicellulose and lignin. Similar results were reported in cellulose nanofiber isolation from fique, where Hoyos et al.^27^ used 5% KOH, 1%NaClO_2_ and 1%HCl as a bleaching medium. The authors demonstrated by FTIR the absence of peaks of lignin and hemicellulose in bleached fibers, however, thermogravimetric analysis showed the presence of shoulders around 300 °C indicating the presence of traces of hemicellulose. On the other hand, the H-F and US-F samples show a more pronounced point of maximum degradation temperature, 340 °C and 317 °C respectively. The degradation temperature of H-F is approximately to DC, while the degradation temperature of US-F is lower than the degradation temperature of DC (338 °C), typical value of cellulose^44^. Our results are close to those reported by Ovalle et al.^26^; who obtained nanofibers from fique residues by the oxidation process with TEMPO combined with ultrasound and whose degradation temperature was 310 °C, temperature lower than the degradation temperature of the cellulose. According to previous reports^34,45^, the low degradation temperatures of the cellulose nanofibers is related to number of free terminal chains which decompose at lower temperatures. Therefore, in accordance with what was reported by Barbash et al.^34^ and Roman and Winter^46^, the isolation of cellulose nanofibers by hydrolysis with H_2_SO_4_ causes a significant decreasing in degradation temperature and an increasing in range of degradation temperature and this is due to the inclusion of sulfate groups in cellulose chains. This behavior was evident in this study. Nevertheless, Rämänen et al.^47^, indicate that the effect of decreasing in thermostability is decreased when sulphate groups were neutralized previously to drying process. The mentioned authors also report that drying method is another factor that influences the thermal stability of cellulose nanofibers, and reports that differences in thermostability are due to fibers reordering due to water elimination. When samples are dried in a conventional manner with air, nanofibers are free to form a homogeneous phase with a defined distance among particles. While drying process through lyophilization requires initial freezing. In lyophilization, nanofibrils cannot be rearranged during water sublimation process, obtaining clusters of dispersed nanofibers and a higher specific surface area^47^, which affects thermal stability. In addition, at low temperatures the fibrils regrouping occurs randomly leaving a large part of free cellulose chains^45^. The above mentioned is important to consider in this study since samples once the hydrolysis process was finished, they were subjected to drying in a forced convection oven and, after ultrasound treatment, they were subjected to drying through lyophilization.

### Particle size

**Figure 4:**
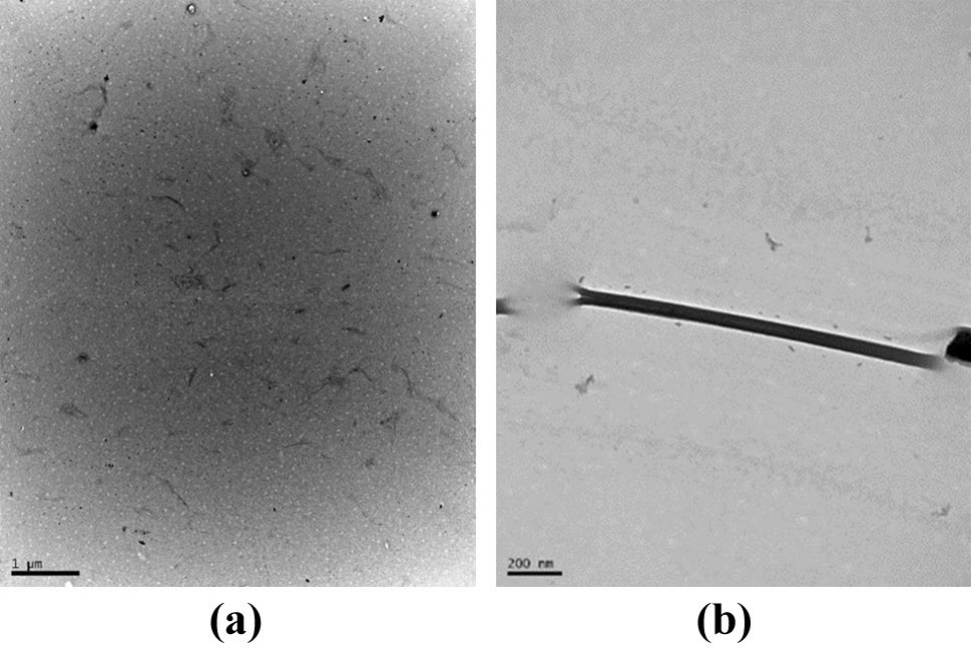
TEM micrographs for fique nanofibers. (**a**, **b**) Scale 1,000 and 200 nm, respectively.

Particle sizes of 25.2 ± 6.2 nm in diameter were obtained for fique nanofibers, which is evidenced in micrographs obtained from TEM analysis (Fig. 4). It is observed that the treatment with H_2_SO_4_ process combined with ultrasound leads to fibers individualization to diameters smaller than 100 nm, which initially ranged from 14 to 21.4 μm. The results of length and aspect ratio (L/D) of the nanofibers were: 483.8 ± 283.2 and 19.2 ± 12.2 respectively.

Treatment with H_2_SO_4_ process combined with ultrasound leads to fibers individualization to diameters smaller than 100 nm, which initially ranged from 14 to 21.4 μm. The results coincide with what was reported by^48,49^ where it is argued that to be considered nanofibers they must have a diameter between 10 to 100 nm while length ranges between 100 and 1,000 nm.

TEM micrograph shows separated and agglomerated nanoparticles. According to what was reported by Song et. al^50^, agglomeration generally depends on Van der Waals attractive forces among nanoparticles. These results are comparable in variability to the report by Trifol et al.^51^ where fique nanofibers production with a diameter of 27.0 nm and length of 658 nm is reported.

On the other hand, the study made by Gomez et al.^27^ reports nanofiber sizes of fiche residues between 8 and 17 nm using bleaching combined with ultra-stex treatment. Similarly, Ovalle et al.^26^, who also worked with fique residues by the method of oxidation by TEMPO and ultrasound, found similar results. While the authors do not specify the diameter of the nanofibers exactly, it is reported that elongated and interlaced structures were obtained, with diameters of around 100 nm and lengths of several micrometers. Although the aforementioned authors used methodologies other than hydrolysis acid, the fiber sizes obtained show low efficiency in the separation of nanofibers from both the amorphous part and the crystalline part of the cellulose structure.

To use nanofibers as reinforcement materials length is an important parameter for determining the diameter ratio (L/D) since the value of this parameter depends on the effect on the mechanical properties on the material to be reinforced. L/D of the nanofibers were 483.8 ± 283.2 and 19.2 ± 12.2 respectively. According to Pelissari et al.^52^, Aspect ratio values ranged from 15.1 to 42.7 can be used as reinforcing agents in composite materials. Therefore, it can be inferred that fique nanofibers can be an alternative for material reinforcement. Likewise, the application of Colombian fique nanofibers for the synthesis of acrylic base hydrogels has not been reported. In addition, nanofibers with an L/D ratio below 60 have the capacity to form a percolated network that is maintained by hydrogen bond interactions, this contributes to an increasing in rigidity and, therefore, fibers with these characteristics have potential as reinforcement using low proportions^53^.

### Strain percent and compression resistance of hydrogel reinforced with nanofibers

**Figure 5:**
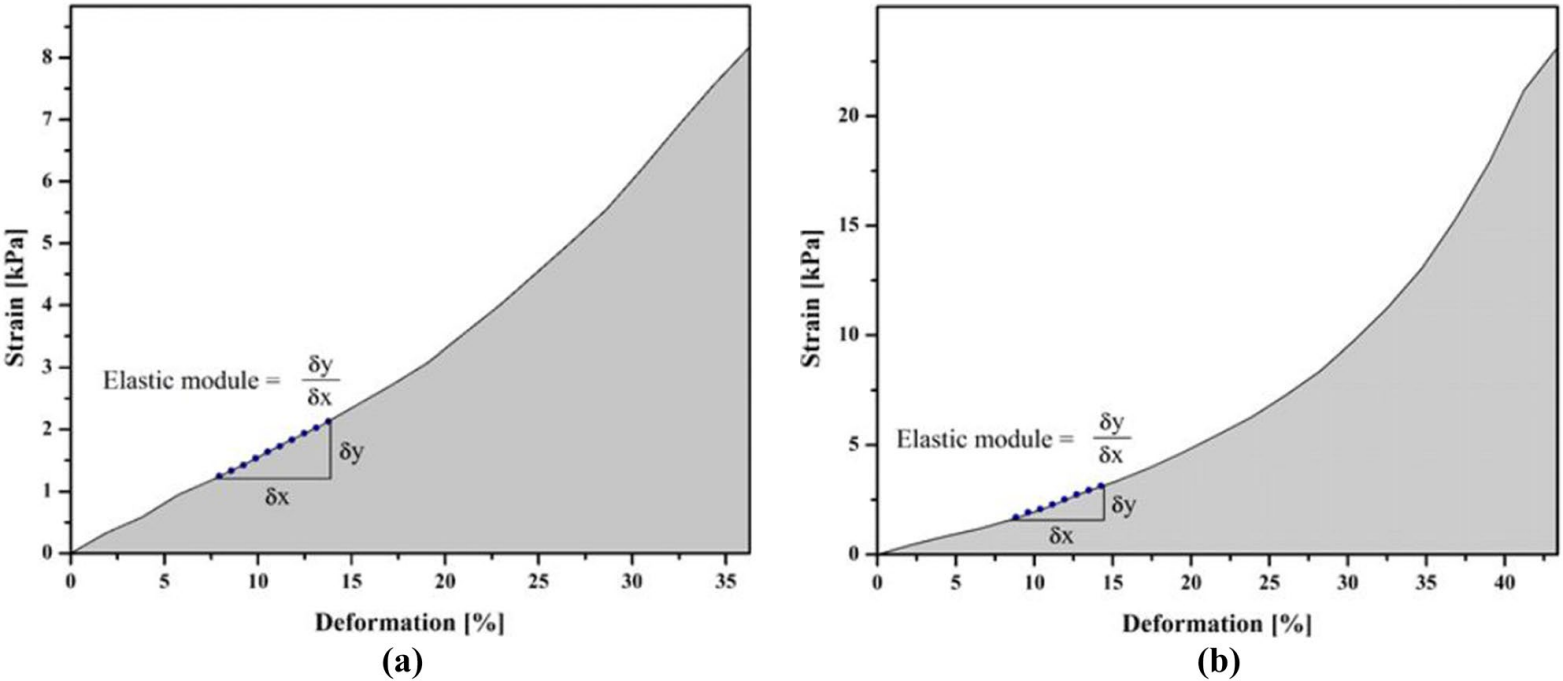
Strain percentage of acrylic-based hydrogels (**a**) hydrogel without addition of fique nanofibers, (**b**) hydrogel with 5% of fique nanofibers.

The Fig. 5 shows the stress–strain curves of hydrogels (H) and hydrogels reinforced with fique nanofibers (HRFN). H presented Rc of 6.49 ± 2.48 kPa while HRFN showed an RC of 16.39 ± 4.30 kPa.

The HRFN supported higher stresses than H because of the HRFN showed higher Rc (16.39 ± 4.30 kPa), and this value is 2.5 times better than the resistance of H (6.49 ± 2.48 kPa). This is explained because the nanofibers interact with the polymer matrix, which favors the stability of the network^54^. Nanofibers dispersed uniformly in the hydrogel help to support higher loads, this could be due to the bonding between the polymer chain and the hydroxyl groups of the nanofibers^11^. Our results are consistent with reports that indicate that concentrations of nanofibers less than 6.7% increase the resistance to compression, higher values of nanofibers in the hydrogel reduce the dispersion of the nanofibers and decrease the crosslinking points^14^. Acrylamide base hydrogels have been synthesized using commercial cellulose nanocrystals of 10 nm in diameter and a length of 120 nm as reinforcement^14^. Similarly, Mahfoudhi and Boufi^10^ obtained acrylate base hydrogels—acrylamide and eucalyptus pulp nanofibers with 5 nm of diameter and 1 mm in length. The addition of these nanofibers increased the stiffness, strength and elasticity of the hydrogel. The authors also reported that at a concentration of 10% nanofibers, the elastic module and compression resistance were up to 10 and 13 times higher than hydrogel without fibers. Our study showed that the addition of fique cellulose nanofibers to hydrogels improves mechanical strength. However, the compression resistance found by Mahfoudhi and Boufi^10^ were considerably higher than our results.

Fique fibers in their native state are characterized by their high specific strength compared to other fibers so it could be expected that there are concentrations of fique nanofibers (different from those added to hydrogels in our research) that can generate mechanical resistances equal to or greater than that obtained by Mahfoudhi and Boufi^10^. This indicates that studies are required to optimize the size and concentration of fique nanofibers added to the hydrogel, and also the use of other methods of pretreating the fibers. Fique nanofibers would be an excellent environmentally friendly option to add value to waste generated in the fique production chain.

**Figure 6:**
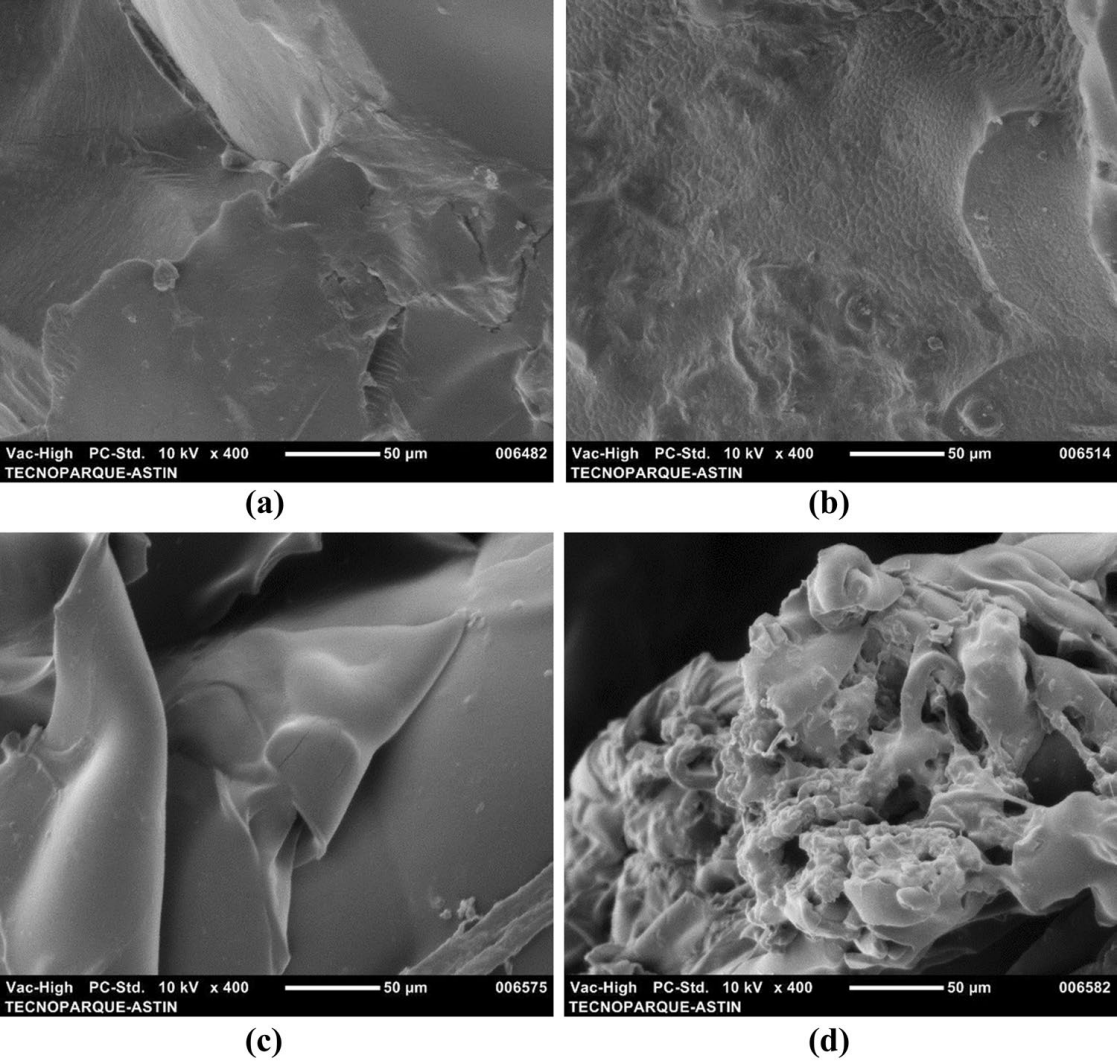
Micrographs (SEM) of acrylic-based hydrogels: (**a**) without nanofibers, (**b**) with 5% of fique nanofibers, (**c**) lyophilized hydrogels without nanofiber, (**d**) lyophilized hydrogels with 5% of fique nanofibers.

Figure 6 shows seM micrographs of H and HRFN at 3% before and after freeze-dried. HRFN shows a rougher surface (Fig. 6b, d) compared to H (Fig. 6a, c). The morphology of HRFN (Fig. 6d) is irregular, with some pores that formed during swelling and subsequent freeze-dried. According to Spagnol et al.^20^, the presence of cellulose nanofibers in hydrogels increases the number of hydrophilic groups, facilitating the diffusion of liquids into the matrix. Consequently, they induce the formation of pores in the structure of the polymer. In fact, the incorporation of nanofibers reduces the crossing points of the hydrogel and dilates the gaps of the network^24^ favoring the penetration of water within the polymer network.

Finally, in this investigation it was evident that the combined process of acid hydrolysis with ultrasound radiation allowed the obtaining of fique nanofibers with diameters less than 100 nm. And that hydrogels reinforced with fique nanofibers considerably improved the compressive strength compared to commercial hydrogels, therefore the nanocellulose obtained from fique has great potential to be used as a reinforcing material in polymeric materials that allows to improve its mechanical properties. However, other research must be done to optimize the concentration of nanofibers needed to maximize the mechanical properties of hydrogels.

## Materials and methods

### Raw material preparation

The raw material was obtained in fique crops at San Bernardo (Nariño, Colombia). The samples were washed with distilled water to remove impurities. The fique was cut into pieces of approximately 1 cm in length. After, fique pieces were dried at 65 °C until constant weight, then were milled in a knife mill and finally sieved to particle size of 0.5 mm (NAT-F).

### Chemical treatment: delignification and bleaching

A mixture of NAT-F and sodium hydroxide aqueous solution (4% w/v) in a ratio 1:25 respectively, was refluxed during 4 h at 90 °C under mechanical stirring, washed several times with distilled water until the alkali was completely removed. Subsequently, the material obtained was bleached using a solution (1:1) of aqueous sodium chlorite (1.7% w/v) and acetate buffer solution (27 g of sodium hydroxide and 75 mL of glacial acetic acid per liter of distilled water). A mixture of delignified fiber and chlorite/buffer solution in a ratio 1:20 respectively, were heated at 80 °C under mechanical stirring and reflux during 6 h. Both, alkali and bleaching treatments were performed in duplicate. Finally, fibers were vacuum filtered, washed until neutral pH with distilled water and dried in a forced convection oven at 50 °C until constant weight^38^.

### Fiber hydrolysis and ultrasound treatment

In a bottom flask, a mixture of 5 g of B-F and 125 mL of sulfuric acid solution (H_2_SO_4_ 6.5 M) was stirred for 24 h at 50 °C under reflux. Subsequently, the mixture was diluted with distilled water and neutralized with NaOH 4% until pH 6.0 to 7.0; the precipitate was filtered and washed with distilled water. For fibrillation process, several suspensions of the obtained cellulose (1 g) were prepared in distilled water (100 mL) and subjected to ultra-turrax treatment at 10,000 rpm during 15 min (H-F). The suspensions (in an ice bath) were homogenized using an ultrasonic homogenizer (BRANSON 1,510), equipped with a ½″ diameter cylindrical probe, at a frequency of 20 kHz with a power of 400 W for 30 min. Finally, the obtained nanofiber suspensions were dried using a freeze-dryer (Labconco, Freezone 6 Plus, USA) operating with a 0.110 mbar vacuum, 48 h, − 40 °C condenser temperature, and 0.03 °C min^−1^ (US-F)^38^. The nanofibers were stored in hermetic containers at 5 °C for further analysis.

### Characterization of raw material (NAT-F), bleached fibers (B-F) and nanofibers (H-F and US-F)

The contents of cellulose and lignin of NAT-F and B-F were determined by the method reported by Va Soes^55^.

### Scanning electron microscopy (SEM)

Surface morphology from NAT-F and B-F were observed in a JEOL table microscope JCM 50000 (Japan). Previously to SEM analysis, samples were coated with gold using the high vacuum mode.

### Infrared analysis (FITR)

Infrared spectra were obtained for NAT-F, B-F, H-F and US-F. Fourier transform infrared (FTIR) spectra were recorded on a spectroscope (Shimadzu, Japan) in a wavelength ranged from 400–4,000 cm^−1^. Commercial microcrystalline cellulose (CC) was used as a control treatment.

### Thermogravimetric analysis (TGA)

TGA were obtained for NAT-F, B-F, H-F and US-F. In addition, TGA analysis of CC was carried out. Thermogram were obtained in a computer DSC/TGA 2STAR system, (Mettler Toledo, USA) with a temperature ranged from 30 to 600 °C at a heating rate of 10 °C/min^−1^, with nitrogen supplying (20 ml min^−1^) and alumina crucibles with approximately 8 mg of sample.

### Diameter of particle

To determine the diameter of nanofibers, images were obtained in a transmission electron microscope (TEM) (JEOL JEM 1,011, Japan). Dispersions of 0.1% by weight nanofibers in water were prepared and mounted on copper grids. Diameters of nanofibers for each sample were estimated using the image processing software (Image J). One hundred measurements were taken to obtain the diameter distribution. The length of nanofibers was determined using Zetasizer (Malvern Instruments) and to measure length, 0.1% by weight suspensions were prepared in distilled water, which were subjected to an ultrasound bath for 30 min. Size measurements were made in quintuplicate, using 1 ml of suspension in a polystyrene cell with a detection angle of 90° at 25 °C.

### Synthesis of hydrogel reinforced with fique nanofibers

Acrylic hydrogels (H) and reinforced acrylic hydrogels with fique nanofibres (HRFN) were synthesized, using the solution polymerization method. Initially, suspensions of fique nanofibers were prepared in 80 mL of water (0 and 5% by weight with respect to the monomer concentration) and dispersed using Ultra Turrax at 9,000 rpm 10 min. On the other hand, 14.0 g of acrylamide (AM) was mixed with 22.0 g of potassium acrylate (AK), obtained by neutralizing 16.0 g of acrylic acid (AA) with potassium hydroxide (KOH). The nanofiber solutions and the monomer solutions (AM and AK) were mixed and stirred during 10 min and subsequently. The initiator potassium persulfate (K_2_S_2_O_8_) (0.3% by weight with respect to the amount of monomers) and the crosslinker N,N-methylene bis acrylamide (NMBA) (0.085% by weight with respect to the amount of monomers) were added to the mixture. Finally, the suspension was refluxed in a 500 mL reactor, equipped with reflux condenser, under nitrogen atmosphere and magnetic stirring. The polymerization reaction was carried out at 70 °C for 6 h. The final polymeric product was cut to reduce the size to approximately 1–2 mm, then washed with ethanol and dried at 70 °C until constant weight^56^.

### Mechanical properties and microscopy (SEM) of hydrogels

The maximum compression resistance (Rc) and strain percent were performed in a universal testing machine (Goobrand 50 IR), according to the methodology proposed by Araki et al.^57^. For this, a 100 N load cell and a 10 cm diameter circular compression plate were used, at a deformation speed of 1 mm min^−1^. The samples were prepared according to what was proposed by Low et al.^58^, the samples were cut in a cylindrical shape, 25 × 25 mm, at their maximum swelling capacity. Strain percent and Rc were measured in triplicate.

H and HRFN al 3% was observed with a scanning electron microscope (JEOL JSM6490) with an acceleration voltage of 20 kV and high vacuum conditions. Additionally, samples were embedded in resin, their water content was extracted by lyophilization and were coated with gold to study their morphology.
