# Author Correction: Valorization of Colombian fique (*Furcraea bedinghausii*) for production of cellulose nanofibers and its application in hydrogels

**DOI:** 10.1038/s41598-020-70781-w

**Published:** 2020-10-14

**Authors:** Marcelo A. Guancha-Chalapud, Jaime Gálvez, Liliana Serna-Cock, Cristobal N. Aguilar

**Affiliations:** 1 National Center for Technical Assistance to Industry (ASTIN), Servicio Nacional de Aprendizaje – SENA, Cali, Colombia; 2 Faculty of Engineering and Administration, Universidad Nacional de Colombia Campus Palmira, Palmira, Colombia; 3 Bioprocesses and Bioproducts Research Group. Food Research Department, School of Chemistry, Universidad Autónoma de Coahuila, Saltillo, Mexico

Correction to: *Scientific Reports* 10.1038/s41598-020-68368-6, published online 15 July 2020.

The original version of this Article contained an error in Affiliation 1, which was incorrectly given as:

“National Center for Technical Assistance to Industry (ASTIN), Servicio Nacional de Aprendizaje – SENA, Medellín, Colombia”

The correct affiliation is listed below:

National Center for Technical Assistance to Industry (ASTIN), Servicio Nacional de Aprendizaje – SENA, Cali, Colombia

This error has now been corrected in the HTML and PDF versions of the Article.

